# Metabolomic Research on Newborn Infants With Intrauterine Growth Restriction

**DOI:** 10.1097/MD.0000000000003564

**Published:** 2016-04-29

**Authors:** Jing Liu, Xin-Xin Chen, Xiang-Wen Li, Wei Fu, Wan-Qiao Zhang

**Affiliations:** From the Department of Neonatology and NICU of Bayi Children's Hospital, The Army General Hospital of the Chinese PLA (JL, X-XC, X-WL, WF, W-QZ); Graduate School, The Chinese PLA Medical College (X-XC), Beijing; and Graduate School, Southern Medical University, Guangzhou (WF), China.

## Abstract

To compare differences in metabolites between newborns with intrauterine growth restriction (IUGR) and those who are appropriate for gestational age (AGA) in order to understand the changes in metabolites of newborns with IUGR and to explore the possible metabolic mechanism of tissue and organ damages in patients with IUGR, with the ultimate goal of providing the basis for clinical intervention.

A total of 60 newborns with IUGR and 60 AGA newborns who were hospitalized in the neonatal intensive care unit of our hospital between January 2011 and December 2015 and who underwent metabolic disease screening were enrolled in this study. The differences in 21 amino acids and 55 carnitines in peripheral blood, as well as changes in the ratios of free carnitine and acylcarnitine to total carnitine, were compared.

Metabolites, particularly alanine, homocysteine, leucine, methionine, ornithine, serine, tyrosine, isovaleryl carnitine, and eicosenoyl carnitine, differed according to newborns’ birth weight (<3rd percentile, 3rd–5th percentiles, 5th–10th percentiles, and 10th–90th percentiles), with those with lower birth weight showing the greater difference (*P* < 0.05). Metabolites also differed by gestational age, and the differences observed were mainly as follows: preterm and full-term newborns showed differences in metabolites, mainly in alanine, proline, cerotoyl carnitine, and tetradecanedioyl carnitine (*P* < 0.05); preterm and full-term AGA newborns showed differences in metabolites, mainly in alanine, glutamine, homocysteine, pipecolic acid, proline, heptanoyl carnitine, and sebacoyl carnitine (*P* < 0.05); and preterm and full-term newborns with IUGR showed differences in metabolites, mainly in arginine, glutamic acid, homocysteine, histidine, leucine, isoleucine, ornithine, serine, threonine, tryptophan, valine, heptanoyl carnitine, decanoyl carnitine, linoleyl carnitine, methylmalonyl carnitine, glutarylcarnitine, sebacoyl carnitine, hydroxyacetyl carnitine, and hydroxyhexadecancenyl carnitine (*P* < 0.05). Among newborns with IUGR, metabolites differed among males and females, mainly in aspartic acid, glutamic acid, and hexacosenoic acid (*P* < 0.05). Birth weight had no significant effects on free carnitine concentration or on the ratios of free carnitine and acylcarnitine to total carnitine (*P* < 0.05).

IUGR infants exhibit significant abnormalities in amino acid and acylcarnitine metabolism, especially those with birth weight below the third percentile. With increasing birth weight, amino acids and acylcarnitines showed compensatory increases or reductions, and when birth weight reached the 10th percentile, the newborns with IUGR resembled the AGA newborns.

## INTRODUCTION

Intrauterine growth restriction (IUGR) refers to the condition in which fetuses do not reach their maximum growth potential in utero; these infants have birth weights lower than the 10th percentile of newborns of the same gestational age.^1^ The incidence of IUGR is 4% to 8% in developed countries and 6% to 30% in developing countries.^2^ IUGR is closely related to high mortality and complications in the fetal and neonatal periods,^3^ and it is also a risk factor for various adulthood metabolic syndromes, such as type II diabetes, coronary heart disease, and hypertension.^4^ In recent years, studies have shown that IUGR is closely associated with fetal–neonatal brain damage, which can easily lead to long-term neurobehavioral abnormalities.^5^ However, studies on the abnormal amino acid and lipid metabolisms of pediatric patients with IUGR remain incomplete. In this study, we planned to use metabolomics to observe changes in peripheral blood amino acid and carnitine concentrations and ratios of free carnitine and acylcarnitine to total carnitine in newborns with IUGR. We hoped to understand amino acid and fat metabolism in newborns with IUGR and clarify whether there were correlated indicators, and if so, which indicators showed metabolic disorders. This understanding would provide a theoretical reference for the further investigation of the mechanisms underlying IUGR-related complications, as well as measures for treatment and prevention.

## SUBJECTS AND METHODS

### Subjects

This study obtained approval from the Ethics Committee of the Army General Hospital of the Chinese PLA. IUGR group: A total of 60 newborns with IUGR (34 males and 26 females; 35 preterm and 25 full-term cases) who were hospitalized in the neonatal intensive care unit of Bayi Children's Hospital affiliated to the Army General Hospital of the Chinese PLA during the period of January 2011 to December 2015 and who met the following criteria were enrolled as subjects: birth weight below the 10th percentile for normal newborns of the same gestational age and gender; underwent of tandem mass spectrometry screening within 3 to 7 days of birth; no genetic metabolic diseases, fetal distress, asphyxia, hypoglycemia, hyperglycemia, jaundice, or brain damage; and no tissue or organ failure. The control group consisted of 60 cases of newborns who were appropriate for gestational age (AGA) (32 males and 28 females; 35 preterm and 25 full-term), who were hospitalized in the same period, and who also met the above criteria.

### Research Methods

#### Measurement Indexes

Amino acids: A total of 21 amino acids were measured: alanine, aspartic acid, arginine, citrulline, glutamine, glycine, glutamic acid, cysteine, homocysteine, isoleucine–leucine, histidine, methionine, ornithine, proline, tryptophan, threonine, serine, tyrosine, valine, phenylalanine, and hexahydropiperidinecarboxylic acid. Acylcarnitines: A total of 55 acylcarnitines were measured: free carnitine, acetyl carnitine, propionyl carnitine, butyryl carnitine, isovaleryl carnitine, hexanoyl carnitine, heptanoyl carnitine, octanoyl carnitine, nonayl carnitine, decanoyl carnitine, dodecanoyl carnitine, tetradecanoyl carnitine, hexadecanoyl carnitine, heptadecanoyl carnitine, stearoyl carnitine, eicosanoyl carnitine, docosanoyl carnitine, lignoceroyl carnitine, pentacosanoyl carnitine, cerotoyl carnitine, methylcrotonyl carnitine, octenoyl carnitine, decenoyl carnitine, sebacoyl carnitine, decatrienoyl carnitine, dodecenoyl carnitine, tetradecenoyl carnitine, tetradecadienoyl carnitine, hexadecenoyl carnitine, oleyl carnitine, linoleyl carnitine, eicosenoyl carnitine, eicosatrienoic dienoyl carnitine, eicosenoic triene acylcarnitine, malonyl carnitine, methylmalonyl carnitine, glutaryl carnitine, adipoyl carnitine, suberyl carnitine, sebacicyl carnitine, docosadiyndioyl carnitine, tetradecanedioyl carnitine, hexadecenedioyl carnitine, linoleyl carnitine, eicosanoyl carnitine, hydroxybutyryl carnitine, hydroxyisovaleryl carnitine, hydroxyhexanoyl carnitine, hydroxydodecanoyl carnitine, hydroxytetradecenoyl carnitine, hydroxyhexadecenyl carnitine, hydroxyhexadecanoyl carnitine, hydroxyoctadecadienoyl carnitine, hexadecanedioyl carnitine, and hydroxyeicosatetraenoyl carnitine.

#### Instruments

We used the Lied Biosystems API 2000 mass spectrometer and Agilent 1100 high performance liquid chromatography for the analyses.

#### Sample Collection

After 6 feeds of sufficient quantity, heel-stick sampling was conducted 3 to 7 days after birth. Blood was added dropwise on special filter paper, and filter papers with dried blood spots were consistently measured.

#### Measurement Methods

Filter papers with dried blood spots were extracted using methanol, which contained internal controls of amino acids and acylcarnitines, and derived using n-butanol hydrochloric acid. The 21 amino acids and 55 acylcarnitines in the peripheral venous blood of newborns were measured using tandem mass spectrometry.

### Statistical Analysis

After data preprocessing, we used SPSS 22 software to analyze serum metabolites of the different newborn groups. Measurement data are expressed using the mean ± standard deviation (x ± s), comparison between 2 groups was conducted using independent samples *t* test, comparison of more than 2 groups was conducted using Levene test for homogeneity of variance, and homogeneity of variance was analyzed using 1-way analysis of variance (ANOVA). Metabolites with significant differences among the groups were screened, and *P* < 0.05 indicated statistically significant differences.

## RESULTS

### Grouping Information and General Information of Pediatric Patients

According to birth weight percentile, the newborns were divided into the following 4 groups: <3rd percentile, 3rd to 5th percentiles, 5th to 10th percentiles, and 10th to 90th percentiles. Somatic parameter and gestational age according to weight groups are presented in Table 1.

**TABLE 1:**
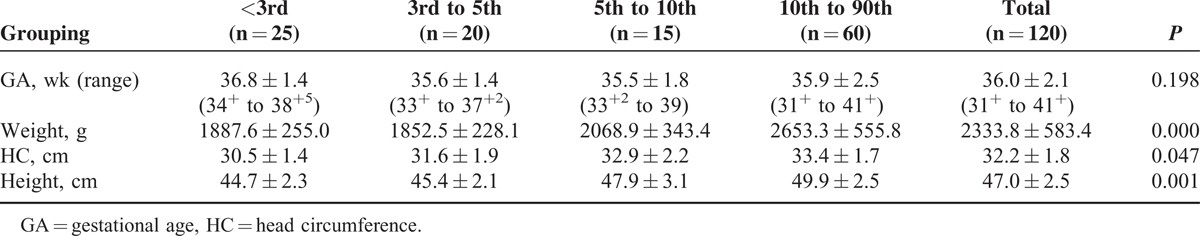
Demographic Information in Different Groups

### Comparison of Metabolites Among Newborns With Different Birth Weight

Table 2 shows the differentially expressed metabolites in newborns of different weight percentages, including alanine, homocysteine, methionine, ornithine, serine, tyrosine, isovaleryl carnitine, and eicosenoyl carnitine (*P* < 0.05). The peripheral blood levels of alanine, homocysteine, methionine, ornithine, serine, and tyrosine were significantly lower in newborns with IUGR weighing less than the 3rd percentile than in AGA newborns. The peripheral blood levels of differentially expressed amino acids showed compensatory increases in newborns with IUGR whose weight in the range of the 3rd to 5th percentiles, and these concentrations were higher than those among AGA newborns, while the concentrations of isovaleryl carnitine and eicosenoyl carnitine increased with increasing weight percentile.

**TABLE 2:**
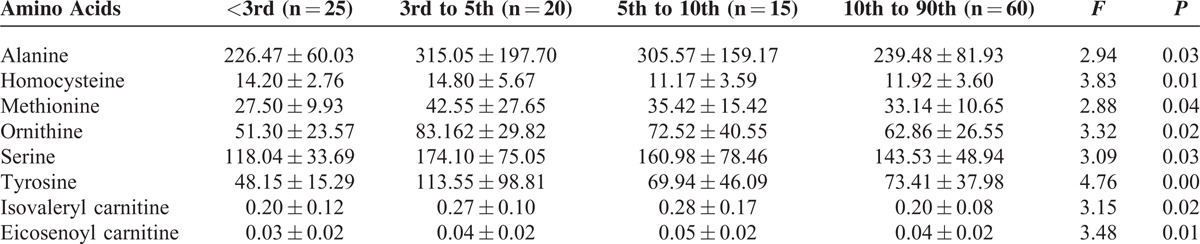
Comparison of the Concentrations of Differential Metabolites in the 4 Groups With Different Birth Weight (μmol/L)

### Comparison of Differences in Metabolites Between IUGR and AGA Newborns by Gestational Age

To clarify whether gestational age can affect various measured factors, we divided the enrolled newborns into 2 groups of preterm and full-term according to gestational age. Based on the weight percentiles, the 2 groups were then further divided into groups of AGA preterm, AGA full-term, IUGR preterm, and IUGR full-term. Pairwise comparisons between groups are shown in Tables 3–5. The results showed that preterm and full-term newborns showed significant differences in peripheral venous blood alanine, proline, cerotoyl carnitine, and tetradecanedioyl carnitine concentrations (*P* < 0.05). The peripheral venous blood concentrations of alanine, proline, and tetradecanedioyl carnitine were higher in preterm newborns than full-term newborns, and the concentration of cerotoyl carnitine was significantly lower in preterm newborns than in full-term infants. Preterm and full-term AGA newborns significantly differed in their peripheral blood concentrations of alanine, glutamine, homocysteine, pipecolic acid, proline, heptanoyl carnitine, and sebacoyl carnitine (*P* < 0.05). The peripheral venous blood concentrations of alanine, glutamine, pipecolic acid, and proline were significantly higher in preterm AGA newborns than in full-term AGA newborns, while those of homocysteine, heptanoyl carnitine, and sebacoyl carnitine were significantly lower in preterm AGA newborns than in full-term AGA newborns. There were significant differences between preterm and full-term IUGR newborns in peripheral venous blood arginine, glutamic acid, homocysteine, histidine, leucine, isoleucine, ornithine, serine, threonine, tryptophan, valine, heptanoyl carnitine, decanoyl carnitine, linoleyl carnitine, methyl malonyl carnitine, glutaryl carnitine, sebacoyl carnitine, hydroxyacetyl carnitine, and hydroxyhexadecenyl carnitine (*P* < 0.05). Of these, the peripheral venous blood concentrations of homocysteine, heptanoyl carnitine decanoyl carnitine, methylmalonyl carnitine, glutaryl carnitine, sebacoyl carnitine, hydroxyacetyl carnitine, and hydroxyhexadecenyl carnitine were significantly higher in preterm newborns with IUGR than in full-term newborns with IUGR. Additionally, the concentrations of arginine, glutamic acid, histidine, leucine, isoleucine, ornithine, serine, threonine, tryptophan, valine, and linoleyl carnitine were significantly lower in preterm newborns with IUGR than in full-term newborns with IUGR.

**TABLE 3:**
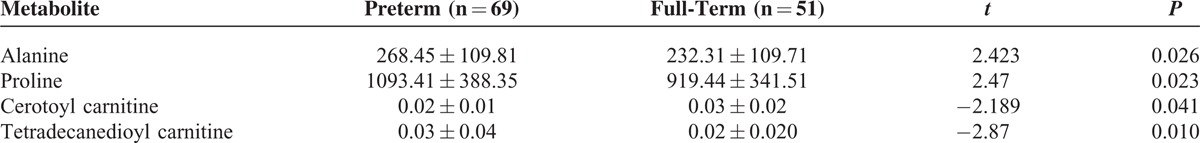
Differential Metabolites Among Preterm and Full-Term Newborns (μmol/L)

**TABLE 4:**
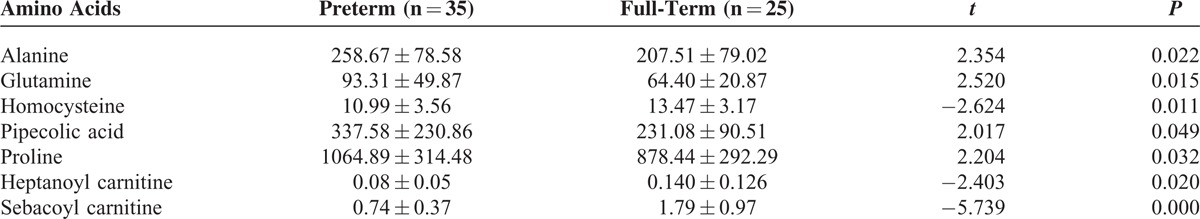
Differential Metabolites Among Preterm and Full-Term Appropriate for Gestational Age Newborns (μmol/L)

**TABLE 5:**
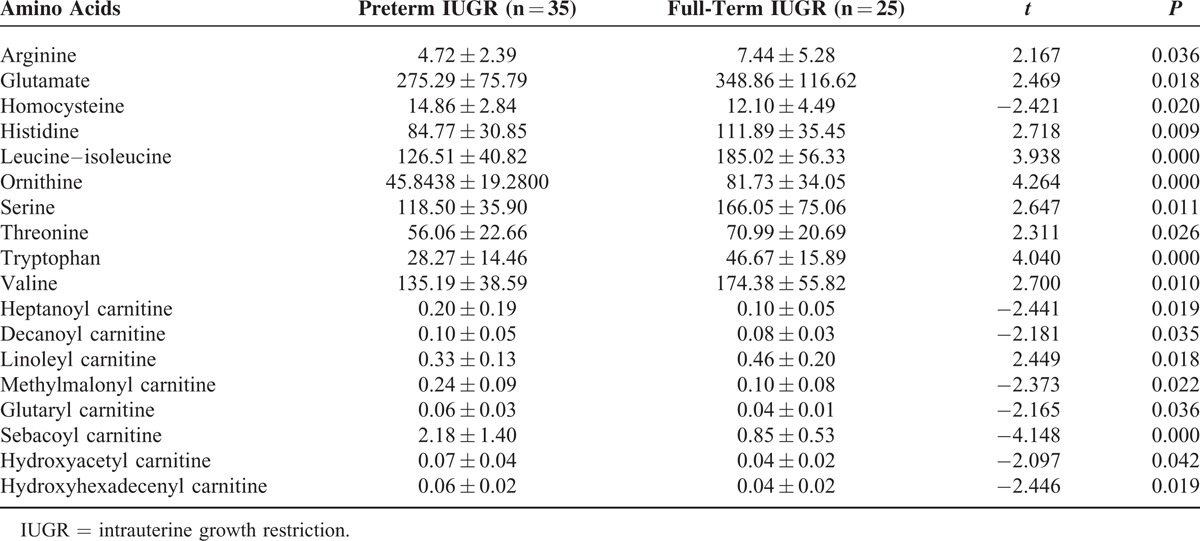
Differential Metabolites Among Preterm and Full-Term Newborns With IUGR (μmol/L)

### Differences in Metabolites Between IUGR and AGA Newborns by Gender

To understand whether gender affects the metabolism of newborns with different body weights, we divided all enrolled neonates into groups based on gender and compared the differences in metabolites between groups. Our results showed that gender did not affect metabolism (*P* > 0.05) among AGA newborns, while gender did affect the concentrations of aspartic acid, glutamic acid, and hexacosenoic acid (*P* < 0.05) among newborns with IUGR, with males having lower concentrations of aspartic acid and glutamic acid and females having higher levels of hexacosenoic acid (Table 6).

**TABLE 6:**
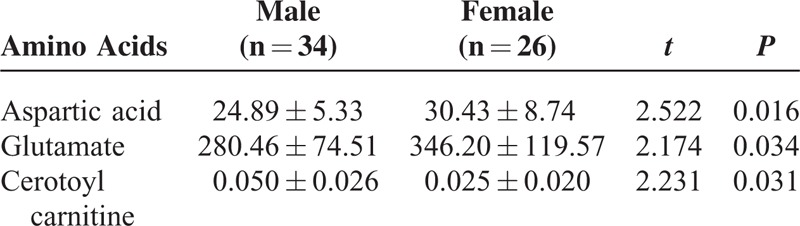
Differential Metabolites Among Male and Female Newborns With Intrauterine Growth Restriction (μmol/L)

### Comparison of the Ratios of Plasma Carnitine and Acylcarnitine in Newborns by Birth Weight

Based on the number of carbon atoms in the acylated groups, carnitines can be divided into free carnitine (C0), short-chain acylcarnitine (C2–C5), medium-chain acylcarnitine (C6–C12), and long-chain acylcarnitine (C14 and above). Carnitines participate in esterification metabolism and can reflect the metabolic state of the human body. The ratio of free carnitine/total carnitine (FC/TC) can indicate the nutritional condition of the newborn, while the ratios of short-, medium-, and long-chain acylcarnitine to total carnitine may represent the status of fatty acid metabolism.^6^ There were no significant differences in the ratios of free carnitine or short-, medium-, and long-chain acylcarnitine to total carnitine among newborns with different weight percentiles (Table 7).

**TABLE 7:**
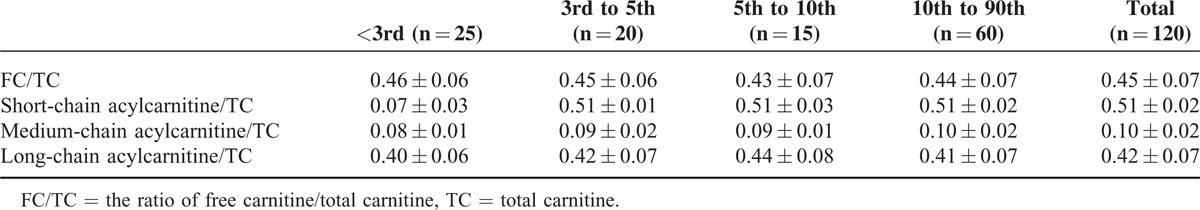
Ratios of Free Carnitine and Acylcarnitine to Total Carnitine in Newborns by Birth Weight

## DISCUSSION

### Metabolic Changes in the Setting of IUGR

Amino acids, carnitine, and acylcarnitine are important for the fetus and are transported from the mother to the fetus through the placenta. They play important roles as the key metabolic factors of the fetus and placenta. It has been shown that fetal metabolism of amino acids, carnitine, and acylcarnitine as well as placental transportation of these factors plays an important role in the pathogenesis of IUGR.^6–8^

Our results showed that birth weight affected the expression of metabolites among newborns with IUGR. Those with a birth weight less than the 3rd percentile showed the most significant differences in amino acids, those with a birth weight in the 3rd to 5th percentiles showed compensatory increases in the concentrations of differential amino acids, and those with a birth weight in the 5th to 10th percentiles showed levels close to those found in AGA newborns. In addition, newborns with IUGR whose birth weight was <3rd percentile showed acylcarnitine levels close to or lower than those found in AGA newborns, and those with a birth weight in the 3rd to 10th percentiles had increased concentrations. Alexandre-Gouaba et al^7^ demonstrated that newborns with IUGR had lower plasma proline, arginine, histidine, and tyrosine levels than the AGA group, which is somewhat similar to our results. There were no significant differences in the ratios of free carnitine and short-, medium-, and long-chain acylcarnitine to total carnitine among newborns with different weight percentiles, suggesting that birth weight does not obviously affect fat and energy metabolism. However, Sanchez-Pintos et al^6^ noted that preterm newborns with IUGR showed differences in fatty acid and energy metabolism within a few days of birth and were of high risk for carnitine deficiency. Our results showed that the in vivo concentrations of differentially expressed amino acids and acylcarnitines in children with IUGR were affected by gestational age and gender. With the exception of homocysteine, the concentrations of differentially expressed amino acids were higher in preterm AGA newborns than among full-term AGA newborns, while the concentrations of differentially expressed carnitines were lower in preterm AGA newborns than among full-term AGA newborns. In contrast, preterm newborns with IUGR had lower levels of differentially expressed amino acids than full-term newborns with IUGR, and with the exception of linoleyl carnitine, preterm newborns with IUGR had lower levels of the other 7 differentially expressed carnitines than full-term newborns with IUGR, including heptanoyl carnitine and decadiene acylcarnitine, which were differentially expressed between preterm and full-term AGA newborns. Gender affected metabolites among newborns with IUGR, but there was no effect among AGA newborns or among all enrolled newborns. These results suggest that gender has limited effects on metabolism in normal newborns within 3 to 7 days of birth, but we cannot exclude the possibility that metabolism may differ according by gender in diseased states (such as IUGR).

### Possible Mechanisms of Metabolite Changes in Newborns With IUGR

#### Mechanisms of Amino Acid Changes in Newborns With IUGR

Compensatory increase in the amino acid concentrations of newborns with IUGR whose birth weight is in the 3rd to 5th percentiles may be caused by a number of factors. First, there may be a reduced utilization rate of amino acids. Studies have shown that newborns with IUGR have altered protein expression profiles, which result in decreased utilization rates of amino acids, which in turn increases amino acid concentrations in vivo.^9^ Second, there may be maternal mechanisms that can compensate for amino acid metabolism in the fetus. Economides et al^10^ suggested that the amino acid concentrations of mothers carrying fetuses with IUGR were higher than those carrying AGA fetuses, and most amino acids were transported from the mother to the fetus through the placenta, leading to compensatory increases in the concentrations of amino acids in the fetus or newborn. Third, there may be compensatory functions of the amino acid transporters in the placenta. The placenta has multiple amino acid transport systems. It can simultaneously transport 1 type or a family of amino acids, and the same amino acid can be transported by multiple transporters. For example, system A can transport neutral amino acids such as alanine, serine, proline, and glutamine,^11^ and leucine can be transported by system L and system b0.^12^ There is mutual compensation and competition between different amino acid transport systems. For instance, serine and glutamate/glutamine absorption can promote the absorption of serine and glutamic acid, while absorption of branched-chain amino acids can actually reduce the absorption of threonine, histidine, and lysine.^13,14^ Newborns with IUGR who weigh less than the 3rd percentile show significantly reduced amino acid concentrations, which may be related to impaired function of the placental amino acid transporters.^12^

By analyzing gestational age, we found that the amino acids that differed between preterm and full-term AGA newborns were completely different from the amino acids that differed between preterm and full-term newborns with IUGR. This difference may be related to the observation that certain amino acids, such as arginine, alanine, aspartic acid, asparagine, glutamate/glutamine, leucine, lysine, and proline, begin to increase only in late pregnancy.^15^

#### Mechanisms Underlying Carnitine and Acylcarnitine Changes in Newborns With IUGR

Newborns with different weight percentiles did not show significant differences in the ratios of free carnitine or short-, medium-, and long-chain acylcarnitine to total carnitine. However, newborns of different weight did show differential isovaleryl carnitine (C5) and eicosanoyl carnitine (C20:1). The levels of differentially expressed acylcarnitine were close or below those of AGA newborns when the birth weight was below the 3rd percentile, and the levels increased when birth weight was in the range of the 3rd to 10th percentiles. The mechanisms underlying the changes in C5 might be due to several factors. First, C5 measurement values included isovaleryl carnitine and 2-methylbut-carnitine, which are derived from leucine and isoleucine, respectively. By analyzing newborns of different weight percentiles, we found that leucine and isoleucine levels decreased in newborns with birth weight below the 3rd percentile, and the levels showed compensatory increases in newborns with birth weight in the 3rd to 10th percentiles (*F* = 3.256; *P* = 0.043). Second, high-risk newborns may receive preventive antibiotic treatment after birth, and antibiotics containing pivalic acid can be converted into pivaloyl carnitine (another C5) in the body, which may interfere with the results.^15^ C20:1 belongs to long-chain acylcarnitine and is associated with the level of fat metabolism and degradation. Its compensatory increase may be related to increased fat metabolism and degradation. When the weight of newborns with IUGR is less than the 3rd percentile, fat storage and this compensatory mechanism are reduced.

### Effects of Metabolic Changes on Tissue and Organ Functions in the Setting of IUGR

#### Effects of Amino Acid Changes on Tissue and Organ Functions

Amino acids, which are the main nutrients in the fetus and newborn, are the basic units constituting proteins; they can maintain nitrogen balance, can be transformed into sugar or fat, and are involved in the structures of enzymes, hormones, and some vitamins. Amino acids are critical for survival and development.^7,17,18^ Abnormal amino acid metabolism tends to lead to issues such as fetal death and IUGR.^19^

Serine and methionine are key components of 1 carbon metabolism and provide methyl groups, thereby promoting DNA synthesis and methylation. Abnormal serine and methionine metabolism in pregnant mothers and their fetuses can cause changes in the enzymes involved in 1 carbon metabolism and increase the risk of uterine and placental insufficiency, leading to IUGR. Uterine and placental insufficiency can also affect 1 carbon metabolism in the liver, as well as DNA methylation, thus changing hepatic gene expressions in children with IUGR. Epigenetic changes in early embryos and the adverse effects caused by IUGR could affect several generations via covalent modifications of DNA and core histones.^20^ Thus, 1 carbon metabolism is essential for embryonic and fetal growth and development, and it can be used to prevent or treat IUGR through epigenetic modifications.^13^

Tyrosine is an aromatic amino acid that can regulate embryonic and fetal neurological development and function, and it is associated with neuronal survival in fetuses with IUGR. Reducing its utilization rate may have permanent effects on fetal neural physiology and postnatal feeding behaviors and life activities.^7,21^

Ornithine is mainly involved in the uric acid cycle and plays an important role in nitrogen discharge. Because newborns with IUGR have reduced uric acid concentrations, the efficiency of the ornithine cycle is decreased, and the liver production of amines is reduced, which affects fetal renal microvasculature and its related functions. Meanwhile, high ornithine may lead to retinal injury.^22^

Alanine is a type of glucogenic amino acids that can facilitate glucose metabolism, help alleviate hypoglycemia, and improve the body's energy. Alanine deficiency tends to cause hypoglycemia, and high alanine leads to risk of gout or diabetes.^23^

Homocysteine concentration increased with decreasing birth weight percentile, which differs from the observation that other differentially expressed amino acids showed compensatory increase in the setting of IUGR among newborns whose birth weight was in the 3rd to 5th percentiles. Homocysteine is mainly transformed from methionine. It has to be kept in low concentrations or in the balanced range in the normal human body, and increased homocysteine concentrations are closely associated with the occurrence of various diseases, such as cardiovascular disease and stroke, osteomalacia disease, brain damage and cognitive decline, oxidative aging, immune system damage, and deficiencies in hormones and vitamins. The higher the concentration, the greater the risk of the related diseases.^24^

Studies have shown that arginine supplementation can reduce the expression of placental apoptotic genes, improve placental function, stimulate the secretion of growth hormone in pregnant women, regulate NO and polyamine synthesis in the placenta, and increase the supply of oxygen and nutrients in the placental blood.^16^ In addition, arginine can also promote the synthesis of placental proteins and the development of fetal intestine through the mTOR signaling pathway, thereby improving IUGR.^16^ Arginine generally exerts its effects in early pregnancy. In this study, we enrolled newborns who were born later than 31 weeks of gestational age, and they showed no significant difference in arginine concentrations. It has been suggested that newborns with IUGR have down-regulated taurine transport systems.^6^ Taurine is essential for fetal development and pancreatic β-cell development, and it can reduce brain cell apoptosis, activate protein kinase signaling pathways to increase neurotrophic factors, and promote brain development in the setting of IUGR.^25,26^ Taurine metabolism is related to alanine, methionine, and cysteine metabolism. Table 2 shows that newborns with different weight percentiles significantly differed in alanine and methionine metabolism. Thus, we do not rule out the possibility that these pediatric patients had abnormal taurine metabolism, which should be further investigated.

#### Effect of Changes in Carnitine and Acylcarnitines on Tissue and Organ Function

Carnitine and acylcarnitine are critical substances for cellular energy metabolism and can be synthesized from lysine and methionine in the human kidneys and liver. Their physiological roles include acting as the sole carrier of long-chain fatty acids and transporting long-chain fatty acids into the mitochondria for β-oxidation and regulating the balance between intracellular free CoA and acyl-CoA. Characteristic changes in 1 or more acylcarnitine indicate abnormal fatty acid β-oxidation and abnormal branched chain amino acid metabolism.^27^

Carnitine plays an important role in the placenta. The fetal placenta has a weak ability to synthesize carnitine, which is mainly supplied to the fetus by the mother. OCTN2 is considered to be a high-affinity placental carnitine transporter, and it can interact with different molecules due to different free radical groups; additionally, it has a very wide range of structural types. Newborns who are small for gestational age (SGA) are considered to be at high risk of fatty acid oxidation disorder caused by carnitine deficiency.^5^ It has been shown that C5 abnormalities are related to metabolic diseases such as isoleucine and leucine metabolism disorders, isovaleric acidemia, and type 2 diabetes.^28^ Among the amino acids, leucine and isoleucine can promote skeletal muscle protein synthesis by activating the mTOR pathway, reduce protein degradation, provide amino groups for mammalian amino acid synthesis, and stimulate the AKT1–mTOR/FRAP1–RPS6K–RPS6 cell signaling pathway during implantation and the perinatal period to promote the proliferation and migration of trophectoderm cells and promote embryonic development.^15^ Abnormal metabolism of leucine and isoleucine can lead to reduced muscle fibers and changes in metabolic pathways of muscle proteins in mid- and late-pregnancy.^29^ C20:1 reduction is also related to the occurrence of type 2 diabetes. It has also been suggested that carnitine can stimulate neuroprotective factors.^30^

## CONCLUSIONS

In summary, the investigation of the overall metabolism of infants with IUGR is still in its early stages. Dessi et al^4^ suggested that intrauterine diagnosis could be achieved through analysis of metabolites and their related ratios, thus clarifying the metabolic status of pediatric patients with IUGR, as well as their potential future complications, which can facilitate targeted therapy. Recent studies have proposed to ameliorate the conditions of IUGR by supplementing amino acids and acylcarnitines, which can help fetuses to reach their maximum growth potential.^31–33^ Our previous study also showed that fetal rat brains affected by IUGR had low taurine levels and that supplementation of taurine in pregnant rats could promote brain development and reduce the incidence of IUGR by several mechanisms^26,34–37^: activating the PKA–CREB signaling pathway, increasing expression of neurotrophic factors, and promoting cell proliferation to counteract neuron loss caused by IUGR; increasing the activity of the PKA–CaMKII/c–fos signaling pathway; reducing neuronal apoptosis in IUGR fetal rats via up-regulating the ratio of Bcl-2/Bax and down-regulating the expression of caspase-3; improving neural stem cell proliferation and differentiation. Thus, the present study provides a reference for further investigation of the mechanism of IUGR and its associated complications, as well as the promotion of fetal development in the setting of IUGR and the improvement of the prognosis via prenatal nutrition intervention.
